# Plasma proteomic profile reveals persistent immune activation in post-acute sequelae of SARS-CoV-2 infection

**DOI:** 10.3389/fimmu.2026.1775044

**Published:** 2026-02-23

**Authors:** Serena Fineschi, Joakim Klar, Jens Schuster, Jonas Bergquist, Niklas Dahl

**Affiliations:** 1 Department of Public Health and Caring Sciences, Faculty of Medicine, Uppsala University, Uppsala, Sweden; 2 Praktikertjänst Health Care Centre Rosendal, Uppsala, Sweden; 3 Science for Life Laboratory, Department of Immunology, Genetics and Pathology, Uppsala University, Uppsala, Sweden; 4 Department of Clinical Genetics, Uppsala University Hospital, Uppsala, Sweden; 5 Analytical Chemistry and Neurochemistry, Department of Chemistry for Life Sciences, BMC, Uppsala University, Uppsala, Sweden; 6 The ME/CFS Collaborative Research Centre at Uppsala University, Uppsala, Sweden

**Keywords:** chronic inflammation, cytokine signaling, immune dysregulation, plasma proteomics, post-acute sequelae of SARS-CoV-2 infection (PASC)

## Abstract

Plasma proteomic profiling of 92 individuals with Post-Acute Sequelae of SARS-CoV-2 infection (PASC), assessed a mean of 34 months after acute infection, revealed a distinct inflammatory signature. Using proximity extension assay technology, 358 proteins were quantified, identifying 26 differentially expressed proteins (DEPs) in PASC: 23 upregulated and 3 downregulated. The most upregulated proteins were Oncostatin M (OSM) and IL-1 receptor antagonist (IL1RN). Additional increases were observed in IL-6, IL-12B, IL-2, CCL22, CSF3, CSF1, and HLA-DRA, as well as proteins involved in tissue remodeling and angiogenesis such as ANGPTL2 and TGFA. Random forest analysis confirmed IL1RN, OSM, ANGPTL2, HLA-DRA, and CLEC4A as strong discriminators between patients and controls. Gene set enrichment analysis demonstrated activation of multiple immune pathways, including Inflammatory Response, TNF-α/NF-κB signaling, IL-6/JAK/STAT3, IL-2/STAT5, and Allograft Rejection, indicating persistent activation of innate and adaptive immunity. STRING network analysis highlighted a tightly connected cytokine-driven inflammatory module. Plasma spike protein levels did not differ between patients and controls, suggesting that PASC-related inflammation may persist independently of ongoing viral replication. Overall, the findings indicate a consistent low-grade inflammatory state in PASC without evidence for distinct biological subtypes.

## Introduction

The COVID-19 pandemic, caused by the SARS-CoV-2 virus, has had profound implications for global health, with a substantial proportion of individuals experiencing persistent symptoms long after recovery from the acute infection. The Post-Acute Sequelae of SARS-CoV-2 infection (PASC), commonly referred to as Long COVID, represent a complex, multisystem syndrome that can persist for months or even years (1). Individuals with PASC report a diverse range of symptoms, including fatigue, cognitive dysfunction, dyspnea, and musculoskeletal pain, which can significantly impair quality of life and lead to long-term disability (2).

Despite a growing clinical recognition, the pathophysiology of PASC remains incompletely understood. The lack of validated molecular markers has hampered the development of both specific diagnostic tools and effective therapeutic interventions. However, accumulating evidence from independent studies suggests the presence of inflammatory marker proteins in plasma of individuals with PASC at various time points following SARS-CoV-2 infection (3–8).

Among the cytokines most frequently reported as upregulated in individuals with PASC are IL-1β, IL-6, and TNF-α (9). While their roles in the “cytokine storm” characteristic of severe acute COVID-19 are well established, there is no consensus that these cytokines are the primary drivers of chronic inflammation in PASC (10).

In a large UK cohort of hospitalized COVID-19 patients (the PHOSP-COVID study), plasma protein profiling at 5 months after discharge revealed that 13 proteins were significantly elevated in post-COVID individuals in the “very severe” recovery cluster compared with the “mild” cluster. These included IL-6, TGFA, CD83, SCGB3A2, CLEC4D, LAMP3, PLAUR, LGALS9, TFF2, EPO, FLT3LG, AGRN and FST (11).

In another longitudinal study of individuals with PASC, followed from 33 to 379 days after a mild COVID-19 infection, approximately 60% exhibited ongoing inflammation, while 40% did not. The inflammatory subgroup was characterized by persistent activation of IL-12/interferon-γ signaling and an NF-κB-driven inflammatory response, possibly initiated by TNF-α and resulting in elevated IL-6 expression (12).

The stratification of PASC into inflammatory and non-inflammatory subtypes has also been proposed by Woodruff and others (13), who identified 12 biomarkers associated with the inflammatory subtype, including IL-6, IL-8, and NF-κB.

Further efforts have been made to identify specific biomarkers in subgroups of PASC (14, 15). Given the heterogeneity of symptoms, several studies have attempted to associate plasma protein profiles with distinct post-COVID phenotypes, including cardiovascular, cognitive, gastrointestinal, fatigue, and anxiety/depression manifestations (14). Notably, children with PASC have shown aberrant plasma protein profiles characterized by elevated expression of pro-inflammatory and pro-angiogenic chemokines such as CXCL11, CXCL1, CXCL5, CXCL6, CXCL8, TNFSF11, OSM, and STAMBP1a (16).

It has been suggested that the chronic inflammation in PASC may be driven by viral persistence, with some studies reporting elevated levels of SARS-CoV-2 spike protein in the plasma of individuals with PASC up to one year or more after acute infection (17–19). However, this association remains uncertain and is still under debate.

Despite the growing number of studies on protein profile in PASC using mass spectrometry and proximity extension assay technologies, there remains a lack of research with extended follow-up, particularly in individuals who were not hospitalized during the acute phase of infection. Considering that certain clinical symptoms improve significantly over time, while others persist, it is important to conduct proteomic analyses in patients with very long-lasting symptoms. Moreover, the selection of a homogeneous study population is crucial to minimize confounding results by mixing individuals who experienced severe acute COVID-19 with those who had mild, non-hospitalized disease.

In this study, we used the Olink Explore 384 platform to profile plasma proteins in a cohort of 92 individuals with not previous hospitalized PASC and 73 matched controls who had experienced SARS-CoV-2 infection during the same time period but had fully recovered. In addition, we measured plasma levels of the SARS-CoV-2 spike protein. The primary aim was to determine whether individuals with PASC exhibit persistent inflammation and proteomic alterations detectable up to three years after the initial infection, and whether these alterations were associated with post-COVID symptoms. A secondary aim was to assess whether plasma spike protein levels were elevated in PASC patients and to explore their possible correlation with symptom profiles.

## Material and methods

### Participants

We enrolled 92 individuals who met the WHO’s diagnostic criteria for post-COVID syndrome (20), along with 73 control participants matched by age and sex. The controls had contracted SARS-CoV-2 during a similar timeframe but recovered fully without any persistent symptoms or complications. All participants were infected with COVID-19 between February 2020 and June 2022, and blood samples were collected during two periods: April–May 2023 and November–December 2024.

Inclusion criteria required participants to be between 18 and 65 years old and to have had a acute COVID-19 infection that did not necessitate hospitalization. Prior to enrolment, each patient underwent a physical examination, radiological imaging, and laboratory testing to exclude alternative causes of symptoms.

Exclusion criteria included a history of malignancy, autoimmune disorders, chronic or active infections, ongoing treatment with corticosteroids or immunosuppressive agents, or pre-existing chronic cardiovascular disease prior to their COVID infection. Medical records were reviewed to obtain participants’ clinical histories and relevant parameters from the time of their COVID illness.

Post-COVID individuals were recruited through the Uppsala post-COVID outpatient clinic and the National Swedish COVID Association. Matched control participants were selected from the Östhammar primary care center and Praktikertjänst Rosendal Primary Care Center among patients who sought medical attention for non-COVID-related symptoms.

Among the post-COVID group, 35 patients contracted SARS-CoV-2 during Sweden’s first wave (February–August 2020), 22 during the second wave (September 2020–February 2021), 13 during the third wave (March–July 2021), and 22 during the fourth wave (August 2021–April 2022). Those infected during waves 1–3 had not been vaccinated. In contrast, participants infected in the fourth wave, which was predominantly driven by the Omicron variant, had received at least two vaccine doses.

All participants had a PCR-confirmed COVID-19 infection, with the exception of ten post-COVID patients and nine controls who contracted the virus early in the pandemic, when PCR testing was limited and primarily reserved for severely ill individuals.

The mean time interval between COVID-19 infection and sampling was 34.0 ± 12.4 months for patients (range 14–58 months) and 32.0 ± 12.9 months for controls (range 11–61 months).

For participants infected during the fourth wave (Omicron), the mean interval was 21 ± 7.4 months for patients (range 14–34 months) and 24 ± 10.6 months for controls (range 11–41 months). Among participants infected during waves 1–3 (pre-Omicron), the corresponding intervals were 39 ± 10.2 months for patients (range 25–58 months) and 37 ± 11.0 months for controls (range 24–61 months).

The study was approved by the Swedish Ethical Review Authority (2021-06852-0160) and conducted in accordance with the Helsinki declaration. All participants gave written informed consent.

### Clinical assessment

The study was conducted in two phases: the first took place in April–May 2023, and the second in November–December 2024 in which both patients and controls were recruited at the same time. During these periods, clinical data were gathered, plasma was collected and frozen at -80C, and participants completed several standardized assessment tools. These included the Fatigue Severity Scale (FSS) (21) for physical fatigue, the Mental Fatigue Scale (MFS) (22) for cognitive fatigue, the Montgomery-Åsberg Depression Rating Scale (MADRS) (23) for depressive symptoms, and the Hospital Anxiety and Depression Scale (HAD) (24) for evaluating both depression and anxiety.

Cut-off scores, adapted to the Swedish population, were as follows. For the FSS (range 0–63), a score of ≥36 indicated significant physical fatigue. The MFS (range 0–42) used a threshold of ≥10 for mental fatigue. MADRS scores (range 0–54) were interpreted as follows: 0–12 indicating no depression, 13–19 mild, 20–34 moderate, and >35 severe depression. For the HAD scale, both the depression and anxiety subscales (range 0–21) used a cut-off of ≥7.

Post-COVID symptom burden was further assessed using a Symptom Severity Score (SSS), which rated 17 symptoms on a 10-point scale (0 = no symptom, 10 = maximum severity), as presented in previous studies (25, 26).

### OLINK inflammatory panel

Plasma samples from the participants were analyzed using the Explore 384 Inflammation assay (Olink Proteomics AB, Uppsala, Sweden) by the analysis service at Olink. The following assays did not meet quality control criteria and were therefore not included in the project: BCL2L11, BID, LTA, PTPRM, RAB6A, CD40LG, IDS, CLEC7A, HGF and MGLL. Data are expressed as normalized protein expression (NPX) values, Olink Protemics’ arbitrary unit, on a log2 scale. NPX values were acquired by normalizing cq-values against extension control as well as an interplate control and a correction factor.

### SARS-CoV-2 Spike Protein analysis

Plasma of 92 patients and 67 controls was analyzed using MSD S-PLEX SARS-CoV-2 Spike kit (Meso Scale Discovery, Rockville, MD). The samples were analyzed according to the manufacturer’s instructions at SciLifeLab Affinity Proteomics (Uppsala University, Sweden). The dynamic range (Limit of Detection (LoD) to highest value) was 104.5 fg/mL to 376–000 fg/mL. For graphing and analysis, any concentrations below the LoD were assigned the LoD value.

### Data analysis

Differences in assessment scores between PASC and control group were evaluated using a two-sample t-test assuming equal variance (p-value cutoff 0.05) or Fisher’s exact test (p-value cutoff 0.05). Linear correlations between severity scores and BMI, as well as severity scores and age, were assessed using the Pearson correlation coefficient and reported as R².

Data from the Olink Inflammatory Panel were analyzed using R (The R Foundation for Statistical Computing, Vienna, Austria) with the R package *OlinkAnalyze* (GitHub: github.com/Olink-Proteomic/OlinkRPackage/tree/master/OlinkAnalyze). Groups were compared using t-tests, and p-values were adjusted for multiple testing using the Benjamini-Hochberg method (adjusted p-value, padj; significance cutoff 0.05). Heatmaps displaying Z-scores for each protein were generated using the R package *pheatmap*.

A random forest analysis, a supervised machine learning approach, was performed to explore the relationships between protein assays and study groups. This method is well suited for capturing complex, non-linear interactions between biomarkers. The analysis was conducted with the primary aim of ranking biomarkers according to their relative importance rather than developing a predictive model. Variable importance was assessed using permutation importance. Internal model validation was performed using the out-of-bag (OOB) procedure inherent to the random forest algorithm, whereby each tree is trained on a bootstrap sample and evaluated on observations not included in that sample. This approach provides an internal estimate of model error and reduces the risk of overfitting without the need for an explicit train/test split or cross-validation. The random forest analysis was implemented using the *ranger* package in R.

Gene Set Enrichment Analysis (GSEA) was performed on the differentially expressed proteins using the Hallmark gene sets from the Molecular Signature Database (MSigDB). Additionally, protein-protein interaction networks were analyzed using *STRING* to explore interactions among the differentially expressed proteins and identify key network hubs.

Further, within the patient subgroup, associations between differentially expressed proteins and age, BMI, symptom severity scores, and log2-transformed spike protein levels were assessed using linear regression. Differences in protein levels between patients infected with the Omicron variant and those infected with earlier variants were assessed with t-tests. P-values were adjusted for multiple comparisons using the Benjamini-Hochberg method.

## Results

### Clinical characteristics of patients and controls

Detailed clinical information and responses to rating scales of each individual study participant are presented in **Supplementary Table S1**. The age range of study subjects was between 21 and 64 years, with a mean age of 44.4 ± 10.3 years in post-COVID patients (range 21–63 years), and 44.5 ± 11.5 years in controls (range 21–64 years) (**Table 1**).

**Table 1:** Clinical characteristics and rating scales.

Variable	Patient	Controls	P -value
Individuals	n=92	n=73	
Gender f (%f)	74 (80.4%)	60 (82.2%)	0.85
Age	44.4 ± 10.3	44.5 ± 11.5	0.98
BMI	26.3 ± 5.4	24.3 ± 3.6	0.96
Sick leave	60 (65.2%)	1 (1.3%)	≤0.001
Infection period			
Wave 1	35 (38%)	17 (23%)	0.09
Wave 2	22 (24%)	18 (25%)	1
Wave 3	13 (15%)	10 (14%)	1
Wave 4 (Omicron)	22 (23%)	28 (38%)	0.06
Symptoms duration (months)	34 ± 12.4	32 ± 12.9	0.2
Symptom duration wave 1-3	39 ± 10.2	37 ± 11.0	0.53
Symptom duration wave 4 (Omicron)	21 ± 7.4	24 ± 10.6	0.27
Post-COVID symptom severity score			
Total score	61.0 ± 26.3	8.7 ± 11.5	≤0.001
Cognitive fatigue	6.8 ± 2.5	1.2 ± 1.8	≤0.001
Physical fatigue	6.6 ± 2.3	1.0 ± 1.7	≤0.001
Headache	4.7 ± 3.2	0.9 ± 1.7	≤0.001
Myalgia	4.5 ± 3.2	0.6 ± 1.3	≤0.001
Palpitations	4.4 ± 3.2	0.4 ± 0.8	≤0.001
Insomnia	4.2 ± 3.3	0.8 ± 1.6	≤0.001
Dyspnea	4.1 ± 2.8	0.3 ± 1.1	≤0.001
Dizziness	3.7 ± 2.7	0.4 ± 1.4	≤0.001
Paresthesia/crawling sensation	3.0 ± 3.2	0.4 ± 1.0	≤0.001
Anxiety	3.0 ± 2.9	0.8 ± 1.4	≤0.001
Depression	2.8 ± 2.8	0.5 ± 1.2	≤0.001
Diarrhea/abdominal pain	2.8 ± 2.9	0.3 ± 1.1	≤0.001
Heaviness in the chest	2.6 ± 2.7	0.2 ± 0.8	≤0.001
Fever	2.3 ± 3.0	0.1 ± 0.7	≤0.001
Tinnitus	2.1 ± 2.8	0.4 ± 1.4	≤0.001
Fainting	2.1 ± 2.6	0.3 ± 1.3	≤0.001
Hyposmia/hypogeusia	1.4 ± 2.8	0.2 ± 0.8	≤0.001
MADRS	15.0 ± 7.2	4.9 ± 5.1	≤0.001
HAD anxiety	6.6 ± 4.1	4.3 ± 3.5	≤0.001
HAD depression	7.0 ± 4.1	1.8 ± 2.4	≤0.001
FSS	55.9 ± 9.5	21.6 ± 10.6	≤0.001
MFS	21.3 ± 11.5	3.5 ± 3.7	≤0.001
Education level			
High education	60 (65%)	57 (78%)	0.47
Secondary education	31 (34%)	15 (21%)	0.18
Primary education	1 (1%)	1 (1,3%)	1
Comorbidity at the time of sampling			
Total	44 (48%)	36 (49%)	1
Allergy/asthma	12	11	0.82
Hypertension	6	6	0.77
Depression/anxiety	8	10	0.45
Hypothyroidism	4	1	0.39
ADHD	5	0	0.071
Anorexia	1	1	1
Diabetes	0	1	0.45

Comparison of clinical characteristics and rating scales in post-COVID patients versus controls. Post-COVID Symptom Severity Score (0-170) includes the 17 following symptoms on a 10 point scale (0= no symptom, 10 max severity). MADRS (depression scale 0-54), HAD anxiety (0-21), HAD depression (0-21), FSS (fatigue severity score 0-63), MFS (mental fatigue score 0-42). Values are presented with mean ± standard deviation (SD). P-values are retrieved using t-test from differences between the groups, or Fisher’s exact test for differences between fractions.

The majority of participants in both groups were females, with 80.4% in the patient group and 82.2% among controls. Patients and controls had a comparable socioeconomic backgrounds and similar levels of education. A high educational level was observed in 65% of patients and in 78% of controls (p = 0.47). Body Mass Index (BMI) was similar across both groups (p = 0.96). Comorbidities at the time of infection were largely comparable between patients and controls, with the exception of ADHD, which was more frequently diagnosed in the patient group (**Table 1**). Most patients reported a good general health and a physically active life prior to their COVID-19 infection. At the time of blood sampling and assessment for post-COVID symptoms, 65.2% of patients were on sick leave.

All administered rating scales revealed significant differences between the patient and control groups. Patients exhibited markedly higher levels of both physical fatigue (FSS: 55.9 ± 9.5) and mental fatigue (MFS: 21.3 ± 11.5) compared to controls (p < 0.001). Depression ratings, assessed using both MADRS (15.0 ± 7.2) and HAD Depression (7.0 ± 4.1), indicated mild depression among patients. HAD scores for anxiety (6.6 ± 4.1) were just below the cutoff. Both depressive and anxiety symptoms were significantly more pronounced in patients than in controls (p < 0.001) (**Table 1**). The post-COVID Symptom Severity Score (SSS; range 0–170) was significantly higher in patients (61.0 ± 26.3) than in controls (8.7 ± 11.5) (p < 0.001). The most debilitating symptoms reported by patients, based on a 0–10 scale, were cognitive fatigue (6.8 ± 2.5), physical fatigue (6.6 ± 2.3), headache (4.7 ± 3.2), and muscle pain (myalgia) (4.5 ± 3.2) (**Table 1**).

There was no correlation between the post-COVID symptom severity score and patients age (R² = 0.0148) or BMI (R² = 0.019) (**Supplementary Figure S1**). A comparison between patients who were infected with the earlier COVID variants (wave 1-3) and patients who contracted the Omicron variant (fourth wave) revealed no significant differences in self-reported post-COVID symptoms (p-value >0.05).

### Differentially expressed proteins in patients vs controls

To determine plasma protein profiles associated with PASC, we performed plasma protein analysis using proximity extension assay technology. The total number of proteins detected in all samples was 358. A principal component analysis (PCA) showed overlapping distributions of PASC and control samples (**Supplementary Figure S2**). The results of the t-test comparisons between patients and controls for all 358 proteins are presented in **Supplementary Table S2**.

**Figure 1:**
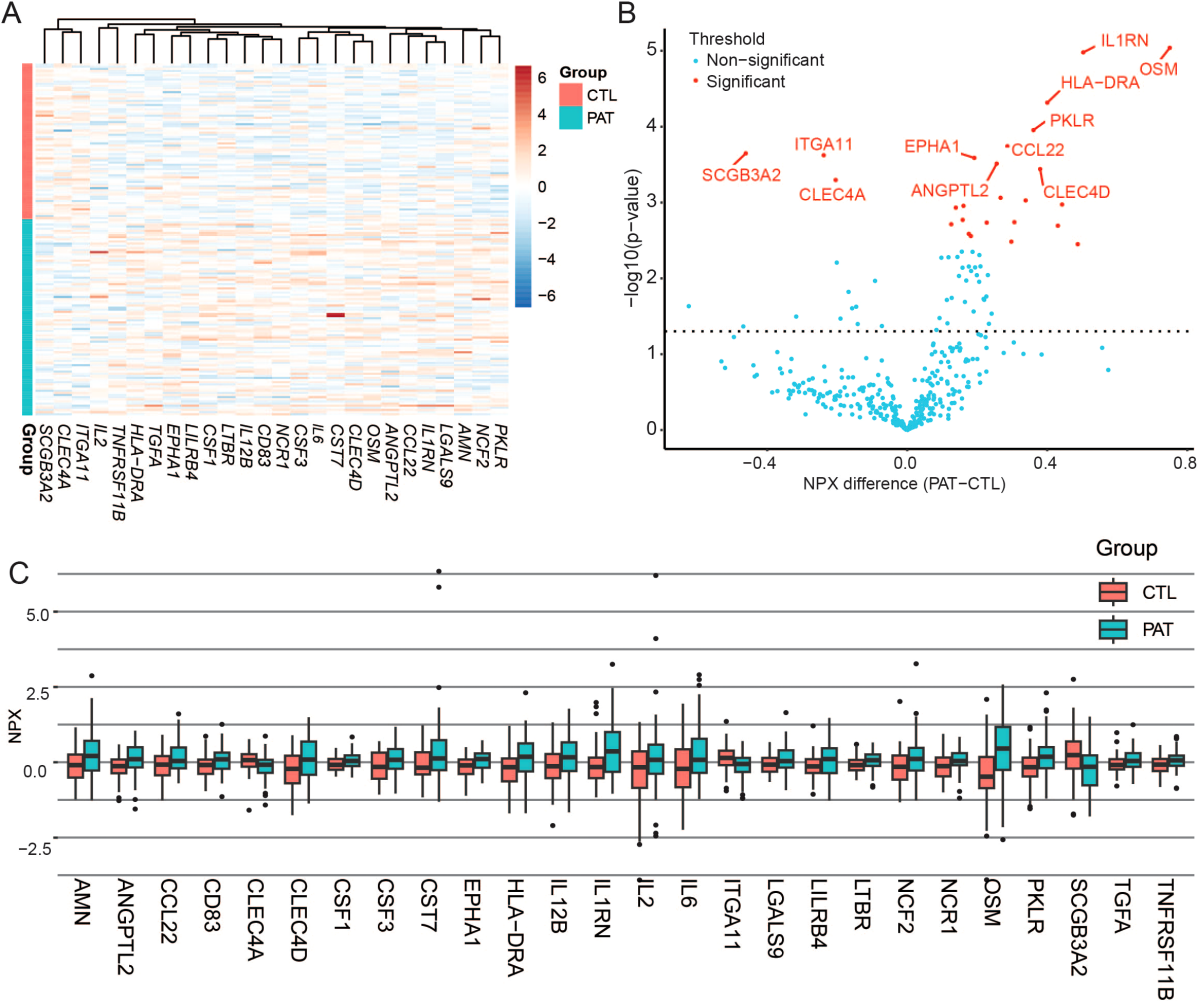
Differentially expressed proteins. **(A)** Heatmap of the differentially expressed proteins between PASC and controls. **(B)** Volcano plots of the p-values from the t-test comparing PASC and controls. The dotted horizontal line represents an unadjusted significance threshold (0.05). Proteins that were significant after p-value adjustment are indicated in red. **(C)** Box-and-whisker plots of expression values (NPX) between patients (PAT) and controls (CTL) of the differentially expressed proteins.

In total, 26 proteins were differentially expressed between patients and controls: 23 were upregulated and 3 were downregulated (**Table 2**; **Figure 1**).

**Table 2:** Differentially expressed proteins between patients (PAT) and controls (CTL).

Proteins	CTL	PAT	P-value	padj	RF Rank
*OSM*	-0.39 (1.00)	0.36 (1.10)	9.1e-06	0.0019	2
*IL1RN*	-0.08 (0.61)	0.42 (0.81)	1.0e-05	0.0019	1
*HLA-DRA*	-0.26 (0.56)	0.14 (0.67)	4.8e-05	0.0058	5
*PKLR*	-0.18 (0.55)	0.18 (0.62)	0.0001	0.0099	6
*CCL22*	-0.13 (0.47)	0.15 (0.49)	0.0002	0.0116	9
*ITGA11*	0.12 (0.40)	-0.12 (0.41)	0.0002	0.0116	29
*SCGB3A2*	0.26 (0.82)	-0.20 (0.72)	0.0002	0.0116	13
*ANGPTL2*	-0.16 (0.38)	0.10 (0.51)	0.0003	0.0122	3
*EPHA1*	-0.14 (0.33)	0.05 (0.33)	0.0003	0.0116	25
*CLEC4D*	-0.26 (0.64)	0.12 (0.70)	0.0004	0.0130	21
*CLEC4A*	0.05 (0.36)	-0.15 (0.38)	0.0005	0.0164	4
*NCR1*	-0.15 (0.41)	0.07 (0.39)	0.0006	0.0171	11
*AMN*	-0.11 (0.54)	0.23 (0.75)	0.0009	0.0240	39
*CSF3*	-0.14 (0.50)	0.13 (0.50)	0.0009	0.0238	8
*CST7*	-0.10 (0.53)	0.35 (1.12)	0.0011	0.0246	35
*TGFA*	-0.08 (0.29)	0.08 (0.33)	0.0011	0.0246	33
*LTBR*	-0.08 (0.25)	0.06 (0.29)	0.0012	0.0246	28
*TNFRSF11B*	-0.09 (0.30)	0.07 (0.33)	0.0017	0.0329	22
*LILRB4*	-0.12 (0.38)	0.11 (0.54)	0.0018	0.0329	12
*NCF2*	-0.18 (0.55)	0.12 (0.69)	0.0018	0.0329	15
*CSF1*	-0.07 (0.24)	0.05 (0.28)	0.0019	0.0329	143
*IL6*	-0.16 (0.87)	0.27 (0.88)	0.0020	0.0329	23
*LGALS9*	-0.06 (0.31)	0.12 (0.42)	0.0026	0.0403	73
*CD83*	-0.12 (0.38)	0.06 (0.38)	0.0028	0.0413	32
*IL12B*	-0.12 (0.62)	0.18 (0.65)	0.0033	0.0470	84
*IL2*	-0.32 (1.02)	0.17 (1.09)	0.0035	0.0488	14

Protein levels are expressed as normalized protein expression (NPX) values (log2 scale) are presented with mean (SD). P-values calculated using t-test were adjusted for multiple comparisons using the Benjamini-Hochberg method. Adjusted p-values (padj) < 0.05 were considered significant. RF Rank is the ranking of the variable importance of the proteins in the random forest analysis.

The most highly upregulated proteins were Oncostatin M (OSM), which belongs to the IL-6 family of cytokines and activates JAK signaling (padj = 0.0019), and interleukin-1 receptor antagonist (IL1RN), which blocks the activity of the pro-inflammatory cytokines IL-1α and IL-1β (padj = 0.0019).

The PASC group showed upregulation of IL-6 (padj= 0.0329), interleukin-12 subunit beta (IL12B) (padj=0.0470), and interleukin-2 (IL-2) (padj= 0.0488), as well as proteins related to tissue repair and angiogenesis, such as Transforming Growth Factor Alpha (TGFA) (padj=0.0246) and Angiopoietin-like protein 2 (ANGPTL2) (padj = 0.0122).

The levels of Colony Stimulating Factor 3 (CSF3), also known as Granulocyte Colony-Stimulating Factor and structurally related to the IL-6 superfamily, and Colony Stimulating Factor 1 (CSF1), also known as Macrophage Colony-Stimulating Factor, were both significantly increased in patients (padj=0.0238 and padj=0.0320, respectively).

Similarly, we detected increased levels of lymphotoxin Beta Receptor (LTBR), a member of the TNF receptor superfamily that strongly activates both canonical and non-canonical NF-κB signaling pathways (padj=0.0246) as well as the Major Histocompatibility Complex Class II, DR Alpha (HLA-DRA) (padj=0.0058).

Furthermore, two molecules that may play a role in reducing inflammation were also upregulated: CD83 (padj=0.0413) and Chemokine ligand 22 (CCL22) (padj=0.0116). CD83, expressed by macrophages, has been described as an important immune checkpoint molecule involved in the resolution of inflammation (27). CCL22 has both pro-inflammatory and anti-inflammatory functions, particularly through its role in recruiting regulatory T cells (Tregs). By promoting interactions between dendritic cells (DCs) and Tregs, it regulates the recruitment of Treg distribution in both health and inflammatory conditions (28).

**Figure 2:**
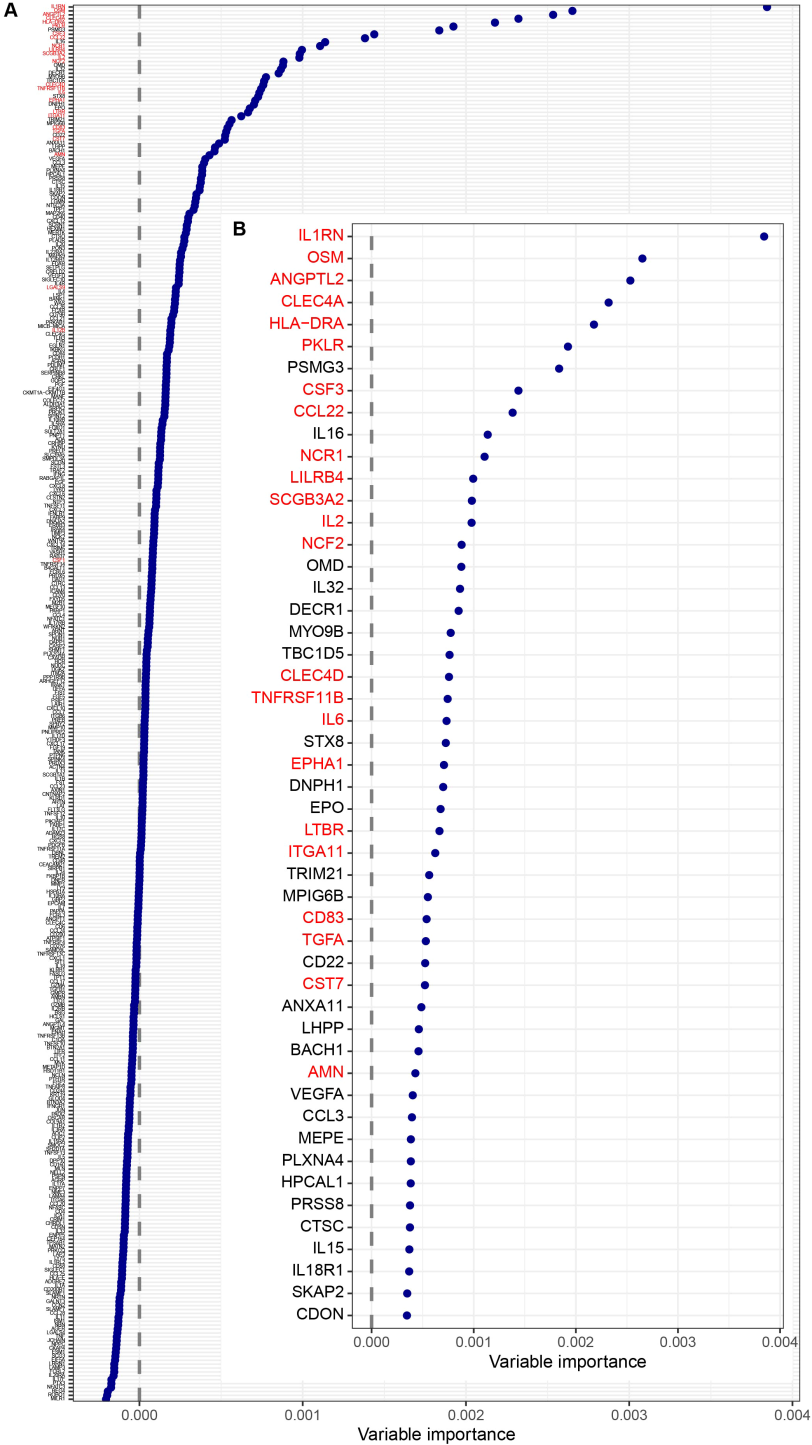
Ranked importance values from the random forest analysis, with the top 50 markers highlighted. The model's c-statistic was 0.753. All 358 proteins are shown on the left **(A)**, and the top 50 are highlighted on the right **(B)**. Proteins that were significant in the t-test comparing patients and controls are indicated in red.

To evaluate the combined discriminatory power of all proteins and to identify key predictors of group classification, we complemented the t-test analysis with a random forest analysis. The proteins with the highest importance in random forest largely overlapped with those from the t-test, supporting the robustness of the findings. The list of ranked importance values, with the top 50 markers highlighted, is provided in **Figure 2**. The proteins with the highest importance for distinguishing PASC patients from controls were IL1RN, followed by OSM, ANGPTL2, CLEC4A, HLA-DRA, and PKLR.

Notably, levels of Proteasome Assembly Chaperone 3 (PSMG3), a chaperone involved in proteasome biogenesis, and the proinflammatory cytokine Interleukin-16 (IL-16), a chemoattractant for CD4^+^ T cells, monocytes, and eosinophils, were not significantly altered in the t-test after adjustment, but showed discriminatory relevance in the random forest model.

### Pathway enrichment analysis (MSigDB) and protein–protein interaction network

Gene Set Enrichment Analysis (GSEA) was performed using the 26 differentially expressed proteins (DEPs) and the Hallmark gene set collection from MSigDB, resulting in five partially overlapping enriched gene sets: Allograft Rejection, Inflammatory Response, TNF-α Signaling via NF-κB, IL-6/JAK/STAT3 Signaling, and IL-2/STAT5 Signaling. All enriched gene sets contained at least three genes and met the significance threshold (padj < 0.05) (**Table 3**).

**Table 3:** Gene set enrichment analysis (GSEA) using the differentially expressed proteins and the Hallmark gene set resulted in five enriched gene sets, padj < 0.05.

Term	padj	Proteins
Allograft Rejection	9.07E-8	NCR1;IL6;CCL22;CSF1;IL12B;HLA-DRA;IL2
Inflammatory Response	1.62E-6	CSF3;IL6;CCL22;CSF1;OSM;IL12B
TNF-alpha Signaling via NF-kB	7.33E-4	CD83;CSF1;IL12B; LTBR; TNFRSF11B; IL6
IL-6/JAK/STAT3 Signaling	8.65E-4	IL6;CSF1;LTBR; OSM
IL-2/STAT5 Signaling	0.0077	CD83;CSF1;CST7**;** IL2; IL12B

Both the Allograft Rejection and IL-2/STAT5 signaling pathways were enriched, reflecting activation of T cells and the adaptive immune response. Within the Inflammatory Response pathway, upregulation of CSF3, IL-6, CCL22, CSF1, OSM, and IL12B was detected, indicating sustained inflammatory signaling. Additionally, enrichment of the TNF-α signaling via NF-κB and IL-6/JAK/STAT3 pathways was observed, consistent with activation of the innate immune system and inflammatory responses.

**Figure 3:**
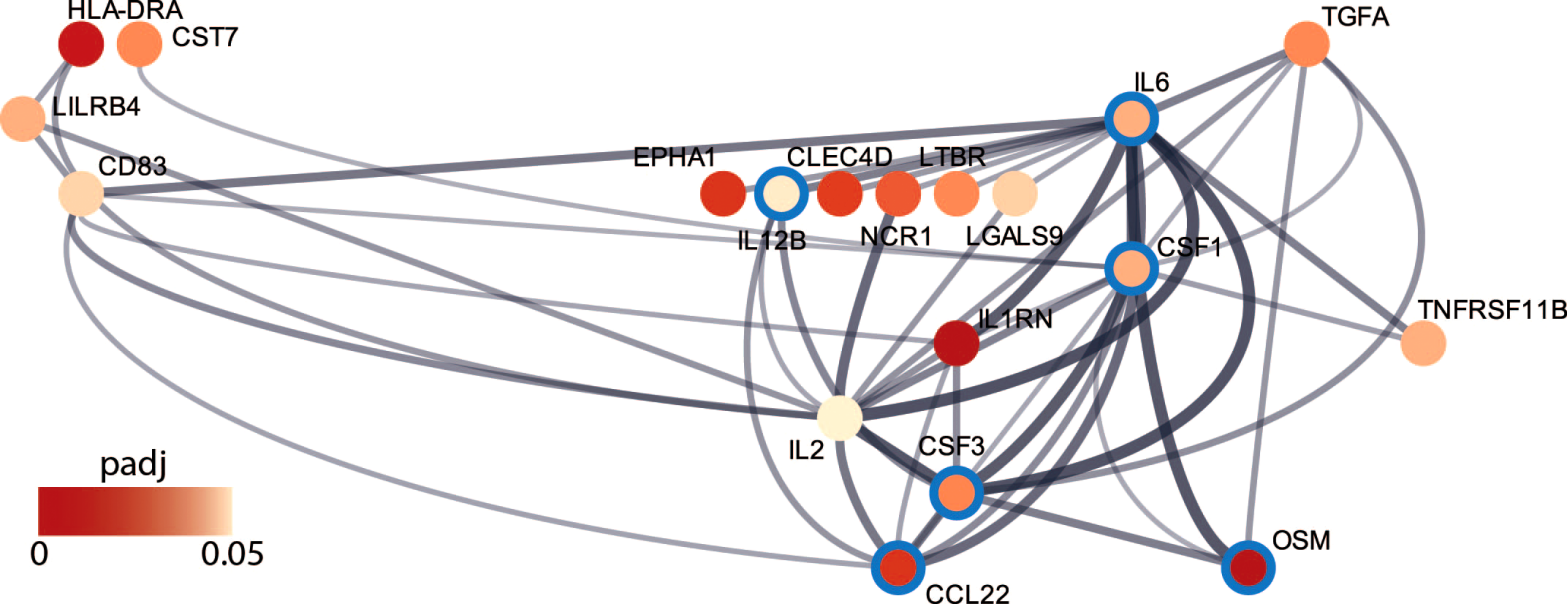
Network from string analysis derived from hierarchical clustering. Proteins belonging to the enriched gene set Inflammatory response is circled in blue. The color gradient inside the circles correspond to the adjusted p-value (padj) from the differential protein expression.

A network analysis using STRING was performed with the differentially expressed proteins to further investigate protein-protein interactions. In total 19 of the 26 proteins created a network with hub proteins belonging to the gene sets Inflammatory response (CSF3, IL6, CCL22, CSF1, OSM and IL12B) from the GSEA (**Figure 3**).

### Correlation between transcriptome analysis and clinical parameters within the group of patients

No significant associations were observed between the 26 DEPs and any of the clinical assessment scores, including MADRS, HAD Anxiety, HAD Depression, MSF, and FSS scores. The Post-COVID Symptom Severity (SSS) total score, as well as each of the 17 individual symptoms included in this scale, also showed no significant associations with the 26 DEPs according to linear regression analysis.

Notably, patients with higher BMI exhibited elevated levels of inflammatory proteins (**Supplementary Table S3**). Linear regression analysis identified positive associations between BMI and 14 of the 26 differentially expressed proteins, including IL1RN, LGALS9, IL6, AMN, LILRB4, ANGPTL2, NCF2, EPHA1, CLEC4D, OSM, CCL22, CSF3, IL12B, and CST7, while SCGB3A2 was inversely associated with BMI.

The comparison between PASC patients infected with the Omicron variant and those infected with earlier SARS-CoV-2 variants revealed no differences among the 26 DEPs (**Supplementary Table S4**). Likewise, no correlations were detected between DEPs and age (**Supplementary Table S5****).**

### Spike protein analysis

**Figure 4:**
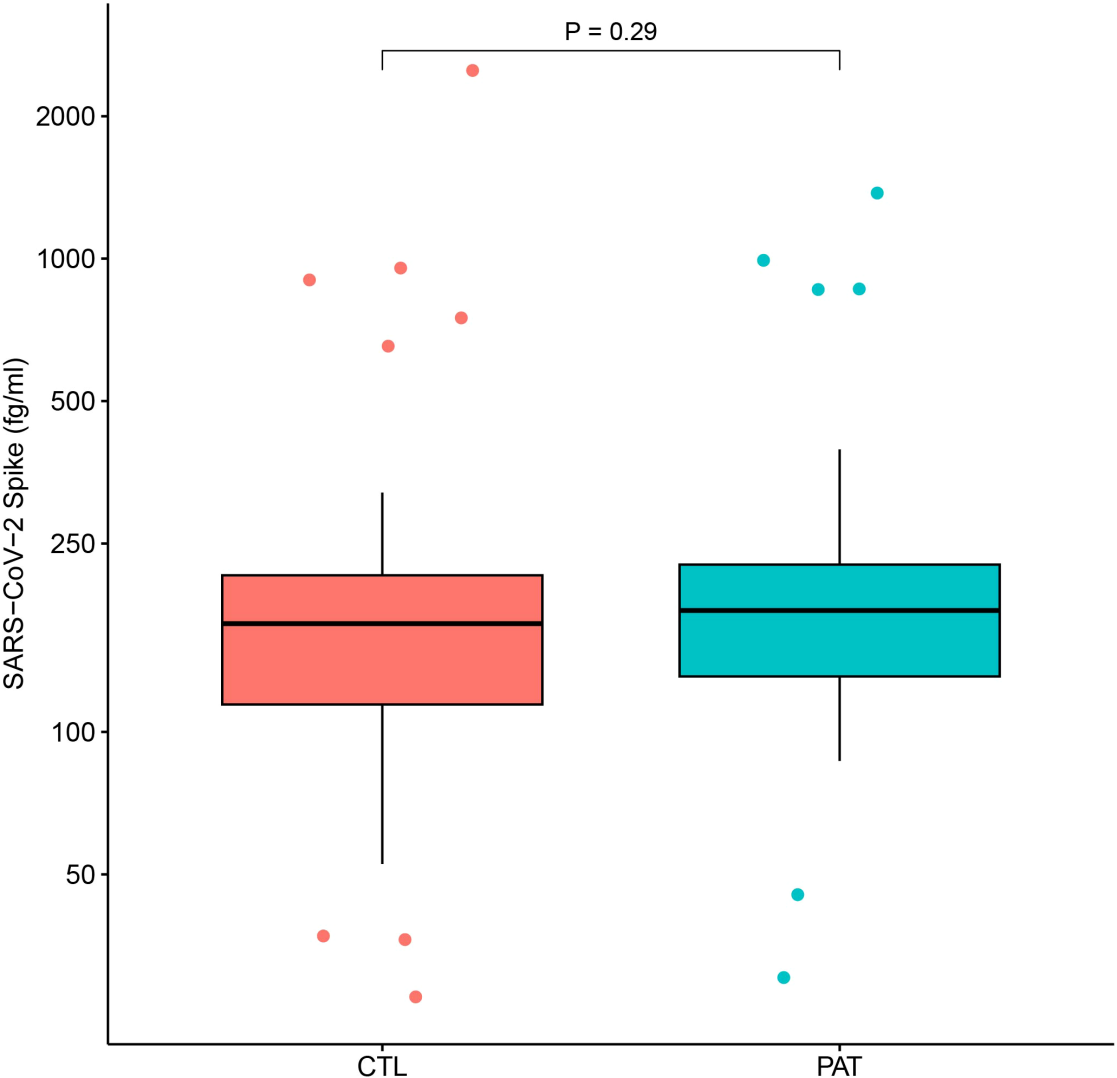
Spike protein level do not differ between patients and controls. Boxplot of log-transformed Spike protein values. The two groups of 92 patients (PAT) and 67 controls (CTL) are compared with Mann-Whitney U test.

We did not detect any significant difference in plasma spike protein levels between patients and controls (Mann–Whitney U test, p = 0.29) (**Figure 4****).** Five outliers were identified in the control group and four in the patient group. None of these individuals recalled having had a recent SARS-CoV-2 infection or COVID-19 vaccination shortly before blood sampling, and the reason for their elevated spike protein levels remains unclear. No correlation was found between spike protein plasma concentrations and the post-COVID Symptom Severity Score, nor with any of the 17 individual symptoms included in this scale. Likewise, linear regression analysis showed no association between spike protein levels and DEPs.

## Discussion

In this study, we demonstrate the presence of chronic inflammation in 92 PASC subjects at a mean follow-up of 34 months after a mild SARS-CoV-2 infection. The homogeneous cohort, together with the very long follow-up time, brings additional important information on the persistent proteomic inflammatory change associated to the post-COVID phenotype.

In a previous study on transcriptome analysis in PBMCs from a cohort of PASC individuals—also included in this study (26)—we reported activation of the JAK/STAT signaling pathway. In the current study, we identified elevated protein levels of OSM, IL-6, CSF3, CSF1, IL-12B, and IL-2, all known activators of the JAK/STAT pathway, consistent with our earlier findings.

Among the 26 DEPs, OSM was the most highly overexpressed (padj = 0.0019) and may represent the primary driver of JAK/STAT pathway overactivation in this cohort of patients. Beyond its role in immune activation and chronic inflammation, OSM is known to contribute to tissue remodeling and fibrosis, promoting fibroblast activation, extracellular matrix deposition, and fibrogenic responses across multiple organ systems (29, 30). Interestingly, elevated OSM levels have been reported both in acute severe SARS-CoV-2 infection and in PASC (31, 32). Russell et al. (31) identified OSM as significantly upregulated in lung tissue from patients who died of acute COVID-19, while a systematic review highlighted OSM as one of the consistently elevated biomarkers in PASC (32). Moreover, a recent study in children with PASC found OSM among the most prominently increased proteins (16). Together, these observations further support a potential role for OSM as a contributing driver of persistent inflammation in PASC.

We also identified additional overexpressed members of the IL-6 cytokine family, most notably IL-6, which plays a central role in both acute SARS-CoV-2 infection and PASC. In severe acute infection, IL-6 contributes to the cytokine storm together with TNF-α and IL-1β. Elevated IL-6 has been described as a strong biomarker of disease severity, correlating with ICU admission, need for ventilatory support, and increased mortality risk (33). Therapeutic agents targeting IL-6 or its receptor—such as Tocilizumab—are widely used to blunt the cytokine storm. Emerging evidence also suggests that IL-6 blockade may dampen long-term immune activation by reducing inflammatory gene expression in innate immune progenitor cells, potentially lowering the risk of developing PASC, though these findings await clinical validation (34). Recent data indicate that early IL-6 elevation, within four days of hospitalization, doubles the risk of developing PASC (35). Moreover, multiple cohort studies have identified persistently elevated IL-6 levels—often alongside IL-1β and TNF-α—for several months after the acute phase of infection, including in individuals who experienced only mild or moderate initial illness (36). Furthermore, IL-6 dysregulation has been especially implicated in neuropsychiatric manifestations of PASC, including fatigue, sleep disturbances, and depression (37). The sustained upregulation of both IL-6 and OSM observed in our cohort up to three years post-infection suggest that the inflammatory response triggered by SARS-CoV-2 can remain unresolved for years, supporting the concept of persistent immune activation as a key driver of long-term PASC symptoms. Our results are consistent with the broader literature and provide further evidence that IL-6–mediated pathways may represent a central mechanism in the pathophysiology of chronic post-COVID conditions, highlighting potential targets for therapeutic intervention.

In addition to OSM, we identified IL-1RA (padj = 0.0019) as one of the two top DEPs in our cohort. By competitively inhibiting the binding of IL-1α and IL-1β to the IL-1 receptor, IL-1RA functions as an endogenous anti-inflammatory mediator. Previous reports have shown that IL-1RA overexpression can mitigate the severity of acute COVID-19 infection (38). The fact that IL-1RA remains upregulated such a long time after infection may reflect a sustained attempt to counteract ongoing IL-1β-driven inflammation.

The third top protein in our random forest analysis was Angiopoietin-like protein 2 (ANGPTL2). This protein has been reported in preclinical studies to contribute to endothelial dysfunction and chronic inflammatory signaling, promoting vascular inflammation, tissue remodeling, and fibrosis, suggesting a potential role in persistent endothelial perturbation in PASC (39, 40).

Among the upregulated enrichment pathways, we found activation of both adaptive immune responses (Allograft Rejection and IL-2/STAT5 Signaling pathways) and innate immune responses (Inflammatory Response, TNF-α Signaling via NF-κB, and IL-6/JAK/STAT3 Signaling). While chronic activation of innate immunity may lead to a pro-inflammatory state—characterized by elevated cytokine levels and persistent activation of monocytes and other myeloid cells—the activation of adaptive immune responses may contribute to T cell dysregulation and autoimmune-like features, including autoantibody production.

We did not observe any correlation between plasma DEPs and post-COVID symptom severity or other clinical parameters, including SARS-CoV-2 variant (Omicron vs. earlier variants). The reasons why clinical symptoms did not align with measurable systemic inflammation in our cohort may be several. Self-reported symptoms such as fatigue, brain fog, or pain can fluctuate over time, and the assessment tools used may not have sufficient sensitivity or may not be fully standardized for this purpose. Single plasma measurements may also represent a limiting factor. Moreover, circulating proteins may not fully reflect tissue-specific damage or organ-specific inflammation, and other mechanisms, such as autonomic nervous system dysregulation, may contribute to persistent PASC symptoms.

Similarly, we did not identify distinct inflammatory subgroups within our cohort of patients. While these findings suggest that symptom patterns may not correspond to distinct inflammatory proteomic signatures, limitations in sample size, statistical power, symptom assessment tools, and natural variation of symptoms over time may have influenced our ability to detect such subgroups. Our observations are consistent with those reported by Talla et al. (12), who also reported no biologically defined inflammatory subgroups in PASC, but do not align with the results reported by Liew et al. (14). Notably, the only significant association identified in our study was between body mass index (BMI) and DEPs, also consistent with observations by Talla et al. (12). Elevated BMI is known to be associated with chronic low-grade systemic inflammation (41), but interestingly we found no association between BMI and post-COVID symptom burden. This suggests that BMI may act as a confounding factor in studies linking PASC to systemic inflammatory signatures, influencing proteomic signatures independently of clinical symptom severity.

Interestingly, the altered plasma protein profile in our PASC cohort partially overlapped with findings from the PHOSP-COVID study (11). That study identified 13 differentially expressed proteins, six of which were also found in our cohort: IL-6, TGFA, CD83, SCGB3A2, CLEC4D, and LGALS9. While the PHOSP-COVID cohort comprised previously hospitalized individuals assessed at 5 months and 1 year post-discharge, our cohort included non-hospitalized PASC patients evaluated up to 3 years post-infection. Together, these findings suggest that similar biological mechanisms may underlie persistent post-COVID symptoms regardless of acute disease severity, and that these mechanisms are likely initiated early and may persist for years.

Persistent viral reservoirs have been proposed as drivers of sustained inflammation in PASC (17–19). However, in our study spike protein levels resulted similar in both patients and controls and did not correlate with DEPs levels or symptoms. This finding does not provide support for the hypothesis that persistent spike protein is a major driver of, or strongly associated with, post-COVID symptoms, but the possibility that viral antigens persist in tissues or at levels below the detection threshold of plasma assays cannot be excluded. Given similar exposure histories to SARS-CoV-2 infection and vaccination in both groups, comparable spike protein levels were expected. The absence of elevated spike levels in PASC does not suggest the presence of ongoing viral replication in plasma, which aligns with our previous findings where we were unable to detect SARS-CoV-2 RNA in peripheral blood mononuclear cells (PBMCs) of individuals with PASC (26). Our results are consistent with a recent study that also found no significant increase in plasma spike protein levels in PASC patients compared to recovered controls, and reported no association between spike protein persistence and symptom severity or functional impairment (42). Taken together, these observations suggest that the inflammatory process in PASC may become self-sustaining and may not require ongoing viral replication in the circulation, though tissue reservoirs cannot be excluded.

A major strength of our study is the long follow-up time of post-COVID symptoms extended over 39 months (mean) in patients who were affected by the pre-omicron variant, accompanied by a detailed clinical phenotyping and comprehensive inflammatory proteomic profiling. Additional strengths include the relatively large number of study subjects and the homogeneity of the patient and control groups regarding ethnicity, socioeconomic back-ground, severity and time of acute infection.

A potential limitation of our study is the focus on a predefined inflammatory protein panel which may have limited the detection of alterations in other biologically relevant pathways, such as metabolic, neurovascular, or mitochondrial processes. Additionally, the cross-sectional design limits causal inference between persistent inflammation and PASC symptoms, and future longitudinal or repeated-measure studies will be needed to strengthen causal conclusions. Finally, our key differentially expressed proteins were not validated using orthogonal methods or independent cohorts in the current study, which should be considered when interpreting their potential biological relevance. Nevertheless, our findings are broadly consistent with previous reports, including the PHOSP-COVID study, with which we share several DEPs, supporting the reproducibility of these proteomic changes.

In summary, our extended proteomic analysis shows that key pro-inflammatory cytokines characterizing the acute phase of SARS-CoV-2 infection remain dysregulated in individuals with PASC, even long after the initial infection. This persistent immune perturbation suggests a failure of proper immune resolution that may not require detectable viral persistence in plasma, although viral antigen persistence in tissues cannot be excluded.

## Data Availability

The datasets presented in this study can be found in online repositories. The names of the repository/repositories and accession number(s) can be found in the article/**Supplementary Material**.
