# Supplementary material for: Plasma proteomic profile reveals persistent immune activation in post-acute sequelae of SARS-CoV-2 infection
Source: Front Immunol. 2026 Feb 23;17:1775044. doi: 10.3389/fimmu.2026.1775044 (PMC12968220; doi:10.3389/fimmu.2026.1775044)

Supplementary Figure S1. Linear correlation between severity score and BMI and severity score and Age assesed with Pearson correlation coefficient.

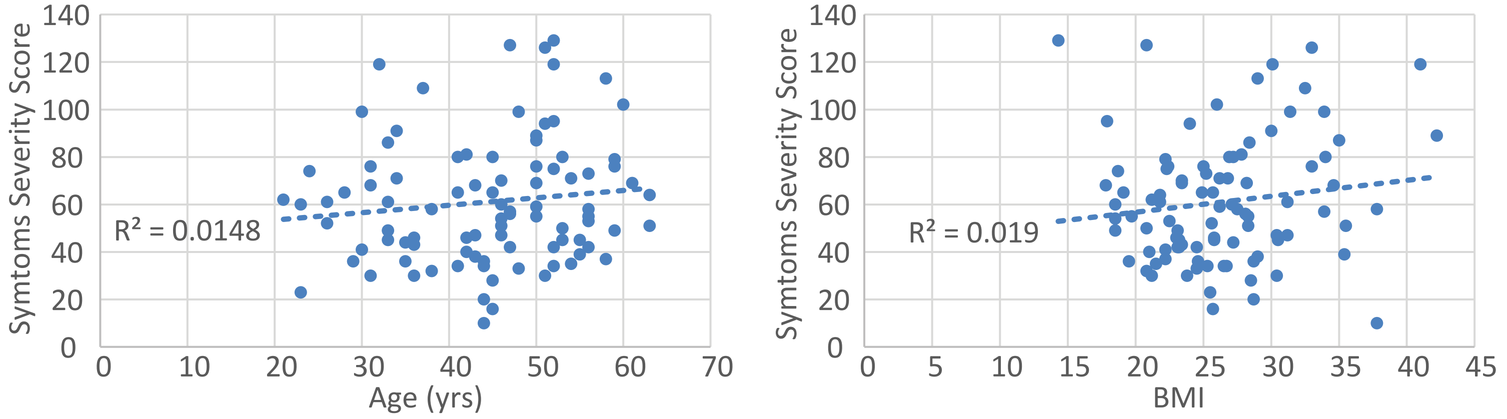

Supplement: Supplementary file 1 [file DataSheet1.pdf]
