# Supplementary material for: Plasma proteomic profile reveals persistent immune activation in post-acute sequelae of SARS-CoV-2 infection
Source: Front Immunol. 2026 Feb 23;17:1775044. doi: 10.3389/fimmu.2026.1775044 (PMC12968220; doi:10.3389/fimmu.2026.1775044)

Supplementary Figure S2. Principal component analysis (PCA) of the 358 proteins, with values centered to zero and scaled to unit variance. The first two principal components are shown with 95% confidence ellipses for each group.

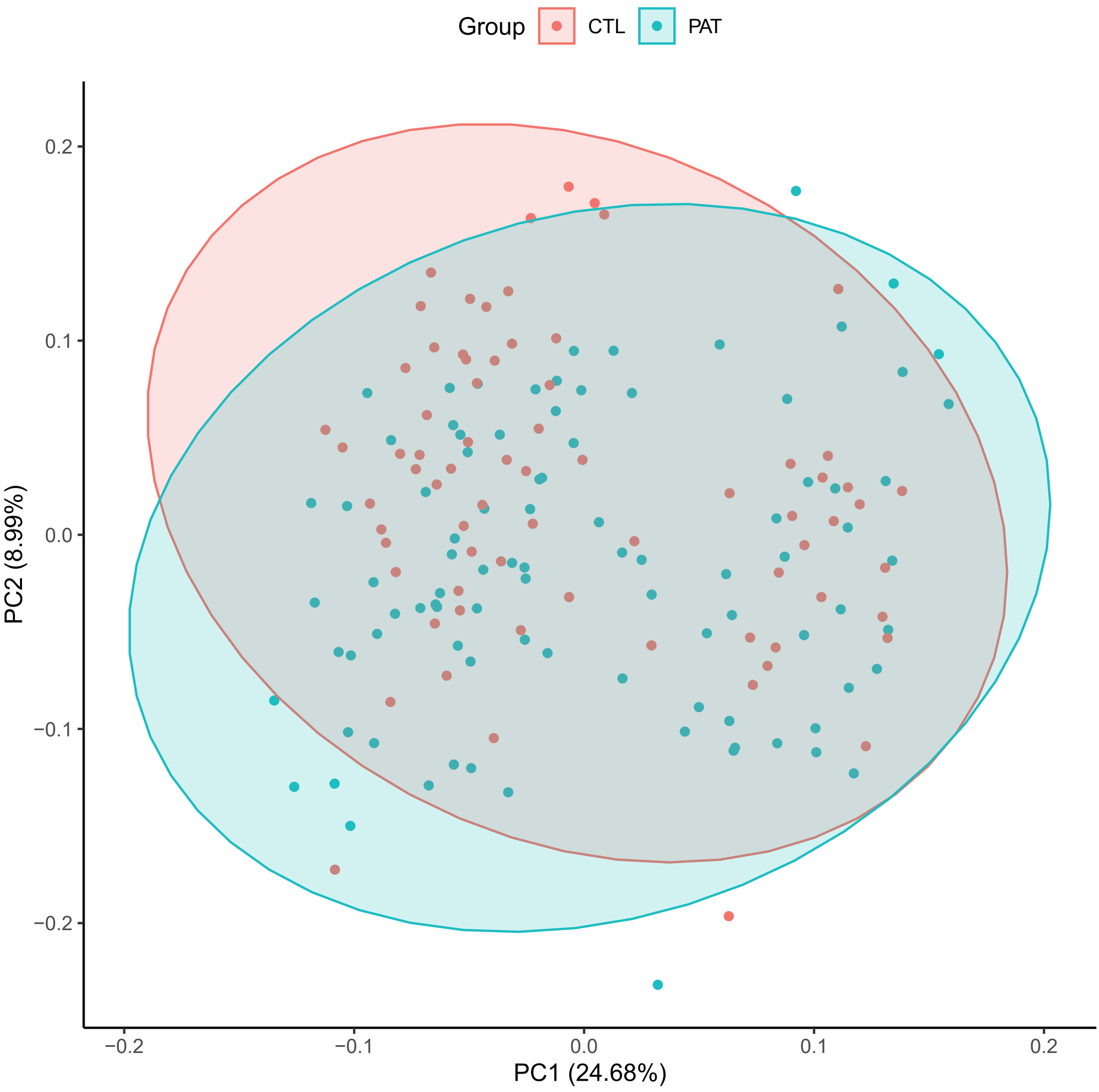

Supplement: Supplementary file 2 [file DataSheet2.pdf]
